# Genetic Distance for a General Non-Stationary Markov Substitution Process

**DOI:** 10.1093/sysbio/syu106

**Published:** 2014-12-09

**Authors:** Benjamin D. Kaehler, Von Bing Yap, Rongli Zhang, Gavin A. Huttley

**Affiliations:** ^1^John Curtin School of Medical Research, Australian National University, Canberra, ACT, 2600, Australia; and; ^2^Department of Statistics and Applied Probability, National University of Singapore, Singapore, 117546, Singapore

**Keywords:** genetic distance, Markov process, maximum likelihood, molecular clock, nonstationary

## Abstract

The genetic distance between biological sequences is a fundamental quantity in molecular evolution. It pertains to questions of rates of evolution, existence of a molecular clock, and phylogenetic inference. Under the class of continuous-time substitution models, the distance is commonly defined as the expected number of substitutions at any site in the sequence. We eschew the almost ubiquitous assumptions of evolution under stationarity and time-reversible conditions and extend the concept of the expected number of substitutions to nonstationary Markov models where the only remaining constraint is of time homogeneity between nodes in the tree. Our measure of genetic distance reduces to the standard formulation if the data in question are consistent with the stationarity assumption. We apply this general model to samples from across the tree of life to compare distances so obtained with those from the general time-reversible model, with and without rate heterogeneity across sites, and the paralinear distance, an empirical pairwise method explicitly designed to address nonstationarity. We discover that estimates from both variants of the general time-reversible model and the paralinear distance systematically overestimate genetic distance and departure from the molecular clock. The magnitude of the distance bias is proportional to departure from stationarity, which we demonstrate to be associated with longer edge lengths. The marked improvement in consistency between the general nonstationary Markov model and sequence alignments leads us to conclude that analyses of evolutionary rates and phylogenies will be substantively improved by application of this model.

A genetic distance measures the number of changes that distinguish biological sequences and is a fundamental metric in molecular evolution. Measurements of genetic distance are employed to address questions pertaining to rates of evolution, the mode and tempo of evolutionary processes, and for some phylogenetic inference techniques. Models that represent sequence evolution as a continuous-time Markov process are the most popular methods for distance estimation. A common assumption made by these models is that the substitution process is stationary, a condition under which the composition of nucleotides or amino acids across the sequences does not change through time. Yet it has long been appreciated that this assumption is often violated (Karlin and Ladunga 1994). Accordingly, alternate metrics that accommodate such compositional heterogeneity across sequences have been developed (Lake 1994; Lockhart et al. 1994). Here we outline the problems with existing methods and propose a new measurement for nonstationary processes. We focus on the implications of the new measure for questions concerning evolutionary rates by considering the existence of the molecular clock.

The development of genetic distances was strongly motivated by observations suggesting a uniform, clock-like accrual of molecular changes in protein sequences through time. The molecular clock hypothesis stimulated major theoretical developments in molecular population and evolutionary genetics, most notably the neutral theory of molecular evolution (Easteal et al. 1995). Estimates of genetic distance are integral to tests for the existence of the molecular clock and to its application of dating historical events.

Errors in genetic distance estimates may contribute to the numerous controversies in the field (Easteal et al. 1995; Kumar 2005). Notable controversies include the reported acceleration of evolutionary rate in the lineage leading to mice compared with that leading to humans (Li 1993; Easteal et al. 1995; Huttley et al. 2007) and that molecular clock dates are systematically more ancient than those estimated from the fossil record (Blair Hedges and Kumar 2003). Although a multitude of biological and geological factors have been identified as potential causes of these discrepancies, the impact of nonstationarity has received very little attention (Kumar and Subramanian 2002). Whether incorrectly assuming reversibility and stationarity could contribute to the frequent rejection of the clock or to discrepancies between molecular and geological dates, therefore, remains unknown.

Before launching into a fuller examination of the approaches for measuring genetic distance, we first define the meanings of the words *process*, *model*, and *submodel*. By process we mean exclusively the probabilistic notion of a stochastic process (e.g., a Markov process). We do not use the term process to refer to a particular history of, or sample from, a stochastic process that we observe in nature, or to mean any sort of computation. In our context, a model is a collection of assumptions about the shape of a phylogenetic tree and the processes that pertain to each of the edges of a tree (e.g., a tree of three taxa, all branching from a single root, with different Markov processes on each edge). Often the adjectives that we apply to a model describe the nature of the processes on its edges (e.g., a Markov model has a Markov process on each edge). A model is a submodel of another if it shares all of the first model's assumptions, but adds some of its own (e.g., the Jukes and Cantor (1969) model is a submodel of the general time-reversible (GTR) model (Lanave et al. 1984)). If we wish to describe a set of samples from a process or model, as we might observe in a multiple sequence alignment, we will describe the sample as being *generated by* the process or model. If we say that one model is *nested* in another, we mean that the model is a submodel of the other.

The idea of using the expected number of substitutions as a genetic distance originated with Eck and Dayhoff (1966), who fitted a reversible Markov model on amino acids to closely related amino acid sequences. Since then, numerous Markov models for nucleotides have been developed, ranging from the simplest model of Jukes and Cantor (1969) to the GTR model (Lanave et al. 1984), as well as models of intermediate complexity and embellishments such as Γ-distributed heterogeneous rates (for review, see Whelan et al. 2001). (Here, and throughout this work, we use the term rate heterogeneity to specify heterogeneity of rates across sites.) According to the model of Eck and Dayhoff (1966), substitutions only happen at regularly spaced time points. However, in most Markov models of nucleotide substitution, substitutions can happen at any time. A continuous-time Markov process is specified by a rate matrix Q, where the off-diagonal entry $Q_{ij}$ is the substitution rate from base i to base j, and the diagonal entries are such that each row sums to zero. Most models of nucleotide substitution are stationary: For a nucleotide sequence undergoing such a substitution process, the nucleotide composition remains roughly the same through time. Given a rate matrix, Q, with strictly positive off-diagonal elements, this stationary distribution is the unique probability row vector π satisfying πQ=0. For a stationary Markov process, the expected number of substitutions in a time interval of length t is equal to

(1) $$n(t)=-\sum_{i=1}^{4}\pi_{i}Q_{ii}t.$$

A simple application of iterated expectations shows that this formula also holds for the standard formulation of Γ-distributed rates. Thus, $n(1)=-\sum_{i=1}^{4}\pi_{i}Q_{ii}$ is the expected number of substitutions in a unit time interval. It has been known at least since Yang (1994) that (1) applies to the GTR model. In all submodels of GTR, (1) reduces to simpler distance formulae. Eck and Dayhoff (1966) obtained an expression very similar to (1), based on the most general time-reversible Markov model for amino acids. Yet it took almost 20 years before the analogous GTR model for nucleotides was developed. A stationary continuous-time Markov process is reversible if for all i≠j, $Q_{ij}=R_{ij}\pi_{j}$, where R is a symmetric matrix. All time-reversible models, including GTR and GTR+Γ, are stationary, by definition. Most commonly used models of nucleotide substitution are time-reversible to reduce the corresponding complexity: Parameters are fewer, and only unrooted trees need to be considered.

We now review the expected number of substitutions as a measure of phylogenetic edge length. Let a and b be nucleotide sequences from extant species sharing an ancestral sequence z, which existed t years ago. Then their phylogeny has the property that the time from z to both a and b is the same: t years. Suppose that the substitution processes on both edges follow a stationary Markov process with rate matrix Q. In most cases, no exogenous estimate for t exists, so that only Qt can be estimated, but not Q. One way around this issue is to consider only calibrated rate matrices, namely Qs for which the expected number of substitutions in a unit time interval is one (i.e., n(1)=1 or $\sum_{i=1}^{4}\pi_{i}Q_{ii}=-1$). Any rate matrix can be calibrated by dividing the rates by n(1). Consequently, both the calibrated rate matrix and the associated edge length can be estimated from data (Felsenstein 2004). Furthermore, the edge length is the expected number of substitutions on the edge, which we now call the genetic distance between the two nodes joined by the edge. A phylogeny whose edge lengths are genetic distances is called a molecular phylogeny.

The notion of genetic distance allows the definition of a molecular clock. In the previous molecular phylogeny, the genetic distance, d, between z and a equals that between z and b, or $d^{za}=d^{zb}$. This is the simplest example of a molecular clock. Suppose that the rate matrix is Q on the a edge, but is kQ on the b edge, where k≠1 is a positive constant. Now we have $d^{za}=kd^{zb}$, so the two distances are unequal: The molecular phylogeny is not consistent with a molecular clock. More generally, a molecular phylogeny with any number of taxa is consistent with a molecular clock if the distance from the root to any taxon is the same; in other words, the tree is ultrametric. We note that this definition of a clock carries over naturally to nonstationary substitution models with an appropriate notion of genetic distance. For stationary models, rate matrices may vary across edges, but they must share the same stationary distribution.

Real homologous nucleotide sequences can frequently exhibit compositional differences. This observation motivated the study of nonstationary substitution models and, in some cases, a corresponding derivation of genetic distance (Yang and Roberts 1995; Galtier and Gouy 1995, 1998; Jayaswal et al. 2005, 2007). Typically, they are similar to the stationary models in having a rate matrix Q, but the initial distribution is not the associated stationary distribution π. Let f(t) be the nucleotide frequency of the sequence at time t. Then f(t) varies with t and converges to π as t tends to infinity. We still use n(t) to denote the expected number of substitutions in the interval [0,t]. Now (1) does not hold. A new formula is needed, giving rise to a new genetic distance.

We now discuss some other approaches to measuring genetic distance that attempt to take nonstationarity into account. Approximations to an expected number of substitutions under very general assumptions exist, such as the paralinear distance (Lake 1994; Gu and Li 1996), but we know of no comparison of this approach with the actual expected number of substitutions for a nonstationary, continuous-time Markov process. Zou et al. (2012) defined the number of substitutions for a discrete-time model (BH: Barry and Hartigan 1987) via a nonstationary GTR-like model that approximates the BH fit. They use a reversible instantaneous rate matrix to represent a nonreversible process, imposing a constraint that is difficult to interpret biologically. It, therefore, remains the case that the impact of employing a reversible process to measure the expected number of substitutions from a nonstationary generating process remains unknown.

In this work, we generalize the genetic distance for stationary processes to nonstationary substitution processes, producing a measure we call expected number of substitutions (ENS). To establish the properties of ENS, we employ a measure of model goodness of fit to identify alignments that are plausibly described by the model under consideration and from which we can, therefore, reliably draw inferences. We include a broad taxonomic diversity and different sequence encoding types by utilizing data from nuclear and mitochondrial protein coding sequences from mammals, and 16S rRNA data from microbes. We show that genetic distances from the GTR and GTR+Γ models and the paralinear distance are consistently overestimated in comparison with ENS. We further demonstrate that the distortions of these popular tools do impact on inference of important biological phenomena, overestimating departures from the molecular clock hypothesis.

## Methods

### Expected Number of Substitutions

Let {X(t):t≥0} be a Markov process on {A,C,G,T} with initial distribution f(0), a row vector, and transition rate matrix Q. Let the equilibrium distribution of the process be π. This substitution process models a single nucleotide in a sequence along one edge of a phylogeny. It is stationary if f(0)=π; otherwise, it is nonstationary. The transition probability matrix over a time interval of length t is P(t)=exp(Qt). The expected number of substitutions in [0,t], denoted by n(t), is

(2) $$n(t)=-\int_{0}^{t}f(s)\text{d}s\text{diag}(Q),$$

where f(s)=f(0)P(s) is the distribution of X(s) and diag(Q) is the column vector consisting of the diagonal elements of Q. This result can be derived by summing over expressions for the expected number of specific substitutions such as on page 154 of Guttorp (1995). For the stationary special case, f(s)=π for any s, so (2) reduces to (1):

(3) $$n(t)=-\int_{0}^{t}\sum_{i=1}^{4}f_{i}(s)Q_{ii}\text{d}s=-\sum_{i=1}^{4}\pi_{i}Q_{ii}t.$$

Note that precisely because f(s)=π, (3) also holds under standard stationary rate-heterogeneous-across-sites models. However, (2) would not hold for a nonstationary rate-heterogeneous model. The extension is straightforward, but as we do not consider such models we will discuss it no further.

We illustrate how the expected number of substitutions can differ from distances obtained from a reversible process using as an example Felsenstein's (1981) model, where $Q_{ij}=\alpha \pi_{j}$ for i≠j, α>0. Since the transition probability matrix has a known form, $f_{i}(s)=\pi_{i}+(f_{i}(0)-\pi_{i})e^{-\alpha s}$, and we have

$$n(t)=\alpha t\sum_{i=1}^{4}\pi_{i}(1-\pi_{i})+\left(1-{\text{e}}^{-\alpha t}\right)\sum_{i=1}^{4}\left(1-\pi_{i})(\pi_{i}-f_{i}(0)\right).$$

If the process is stationary, we get the well-known formula $n(t)=\alpha t\sum_{i=1}^{4}\pi_{i}(1-\pi_{i})$. Thus, the second term above is a correction for nonstationarity. If π is the uniform distribution, then the correction is 0 regardless of f(0), which is well known in the special case of the Jukes and Cantor (1969) model, and also holds for Kimuras two- and three-parameter models (Kimura 1980, 1981). Otherwise, the correction can be positive or negative. Let π=(0.5,0.2,0.1,0.2), α=1.0, and t=1.5. Then for the stationary model, n(t)=0.99. For the nonstationary model with f(0)=(0.1,0.3,0.2,0.4), it is 1.09. If f(0)=(0.6,0.2,0.1,0.1), it is 0.97. Therefore, under a nonstationary process, a naïve application of (1) can over- or understate the genetic distance. Note that we make no representations about the biological realism of any of these scenarios, and leave claims of systematic bias to our empirical findings.

### The ENS Distance

Consider a rooted phylogeny on which operates a substitution process specified by an initial distribution at the root and edge-specific rate matrices. As usual, assume that given the state at an internal node, the processes on the edges below it are independent. For a pair of adjacent ancestor-descendant nodes (μ,ν), let the distribution of $X^{\mu }$ (the state at μ) be $\pi^{\mu }$, the rate matrix be $Q^{\mu \nu }$, and the time between the nodes be $t^{\mu \nu }$. Then the expected number of substitutions from μ to ν is given by $n(t^{\mu \nu })$ in (2), where $f(0)=\pi^{\mu }$ and $Q=Q^{\mu \nu }$. The ENS distance between μ and ν is defined as $n(t^{\mu \nu })$, and is denoted $d_{\text{ENS}}^{\mu \nu }$. The ENS distance between any two nodes is defined as the sum of ENS distances over the edges on the path between them. Our formulation of ENS (2) is similar to one by Simon Tavaré (personal Communication).

For comparison, the expected number of substitutions, if calculated under a GTR model, is written $d_{\text{GTR}}$. Likewise, we denote the expected number of substitutions under GTR+Γ as $d_{\text{GTR}+\Gamma }$. As noted in the Introduction, (1) suffices for calculating either.

Further, the paralinear distance between μ and ν is denoted $d_{\text{para}}^{\mu \nu }$ and defined as follows. Let $P^{\mu \nu }=\exp(Q^{\mu \nu }t^{\mu \nu })$ be the transition probability matrix from μ to ν, $\pi^{\nu }=\pi^{\mu }P^{\mu \nu }$ be the distribution of $X^{\nu }$, and $J^{\mu \nu }=\text{diag}(\pi^{\mu })P^{\mu \nu }$ be the joint distribution of $\left(X^{\mu },X^{\nu }\right)$. The paralinear distance between μ and ν is

(4) $$\begin{array}{l} d_{\text{para}}^{\mu \nu }=-\frac{1}{4}\ln\det\left(\text{diag}{\left(\pi^{\mu }\right)}^{-\frac{1}{2}}J^{\mu \nu }\text{diag}{\left(\pi^{\nu }\right)}^{-\frac{1}{2}}\right)\\ =-\frac{1}{4}\ln\det\left(\text{diag}{\left(\pi^{\mu }\right)}^{\frac{1}{2}}P^{\mu \nu }\text{diag}{\left(\pi^{\nu }\right)}^{-\frac{1}{2}}\right). \end{array}$$

It is important to note that the paralinear distance was intended as an approximation to the expected number of substitutions that can be calculated purely from the joint probability distribution of states at adjacent nodes. For the purpose of comparison, the similar LogDet transformation is defined $d_{\text{LogDet}}=-\ln\det\left(J^{\mu \nu }\right)$. The LogDet transformation “does not estimate the lengths of edges” (Lockhart et al. 1994), so we restrict our analysis to the paralinear distance.

### Statistical Methods and Algorithm Implementation

In this study, all model fitting was performed using PyCogent (Knight et al. 2007). Sequences of nested, continuous-time Markov models were fitted using maximum likelihood (ML) in order of increasing generality to triads of taxa. Where applicable, the initial parameter estimates for a model were obtained from the previous model. Sequential fitting ensures that richer models obtain greater log likelihood. Upper limits were placed on substitution rate parameter estimates. The fitting algorithm was deemed successful if the model converged. Convergence did not fail for any of the fits that we attempted.

Models of rate heterogeneity across sites were assumed to have Γ-distributed rates in four bins. An upper limit was placed on the shape parameter for the Γ distribution. Note that a rate-heterogeneous model with more than one bin and a finite shape parameter is not nested in and does not nest any rate-homogeneous model.

**Figure 1.**
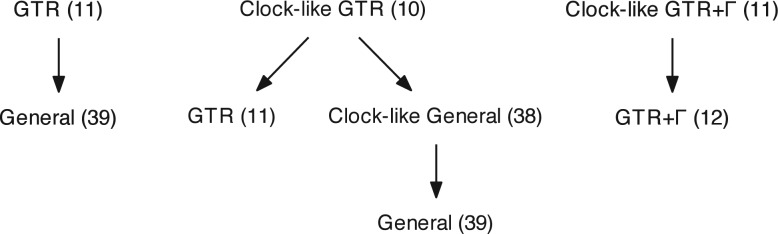
Nested models used for sequential fitting. Arrows point in the direction of generalization, coinciding with the flow of optimization parameter seeding. Parenthesized numbers show the degrees of freedom for the model fitted to a triad of sequences. The first hierarchy was used in most instances. Those including clock-like models were used only for molecular clock testing.

We designate the three types of models that we fitted as *General*, *GTR*, and *GTR*+Γ. The GTR model is a submodel of the General model. The order of sequential fitting is illustrated in Figure 1. The General model places no constraints on the rate matrix on each edge or on the probabilities of states at the ancestor node, so can be nonstationary. The GTR model uses a common, time-reversible rate matrix across all edges and shares the same state probability vector at every node. The common rate matrix constraint for GTR was chosen to reflect common practice in the field. Models labeled as *clock-like*, which were used in the relative rate tests of the molecular clock (Wu and Li 1985), constrain the ingroup edges to be the same length (by the ENS distance). In all other cases, edge lengths were allowed to vary by edge. GTR+Γ models share the same constraints as their GTR counterparts, with one additional degree of freedom which is the rate shape parameter and is common through the tree.

It is common to describe the hierarchy of substitution models that nest in GTR via the way in which they parametrize Q (e.g., Felsenstein 2004, pp. 196–206). We include a possible parametrization of Q for the General model here for the purpose of comparison with such characterizations:

$$Q=\left(\begin{array}{cccc} -\alpha -\beta -\gamma & \alpha & \beta & \gamma \\ \delta & -\delta -\epsilon -\zeta & \epsilon & \zeta \\ \eta & \theta & -\eta -\theta -\iota & \iota \\ \kappa & \lambda & \mu & -\kappa -\lambda -\mu \end{array}\right).$$

**Figure 2.**
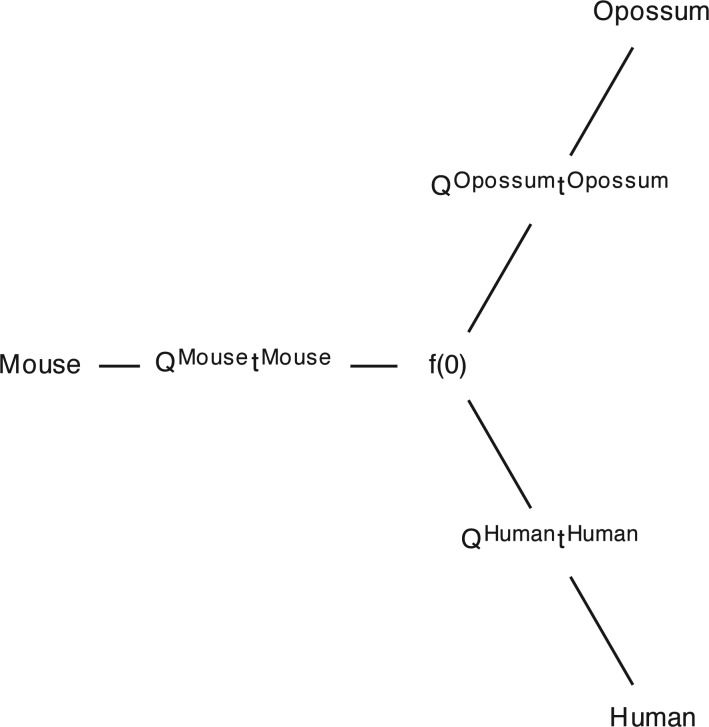
The General model allows Q and t to vary between edges and independently of the starting marginal probability vector f(0).

We also show the variation of Q by edge for the General model in the example topology in Figure 2.

Due to the difficulty of identifying parameters associated with the two edges emanating from the root, these two edges are considered as a single edge, and the fitted process treats the internal node on this new edge as the root. This simplification entails loss of information on the two said edges, but not on the others belonging to the ingroup.

Under mild conditions, ML fits of a Markov model to triads of taxa are consistent (Chang 1996). One of the conditions is that the model is identifiable, which is not always the case for the General model, for two reasons. (We note that identifiability of the GTR+Γ model for triads has been established see e.g., (Allman et al. 2008)).

First, states at internal nodes can be relabelled without affecting alignment column frequencies. This issue is explained in detail in Zou et al. (2011) for the BH model, but is applicable here by Remark 2 in Chang (1996). The issue can be resolved through imposing the constraint suggested by Remark 9 in Chang (1996), which is that the fitted transition probability matrices be Diagonal Largest in Column (DLC).

Second, multiple continuous-time processes can be consistent with a single discrete-time process (Higham 2008). That is, it is possible for valid transition rate matrices $Q_{1}$ and $Q_{2}$, where $Q_{1}\neq Q_{2}$, to give rise to the same transition probability matrix so that $e^{Q_{1}}=e^{Q_{2}}$. In fact, one transition probability matrix can correspond to zero, one, several, or a continuum of transition rate matrices. It is possible to conservatively identify cases where more than one valid transition rate matrix exists using Theorem 1.27 in Higham (2008). We call any such case a nonunique mapping.

Any fits that did not satisfy the DLC and unique mapping constraints were rejected. Both tests are offered in the latest version of PyCogent. Fits that failed the DLC constraint were rare, representing ∼0.36% of a data set in the worst case. Nonunique mappings were also rare, only appearing in the 16S rRNA data set (see the Data Sets section) and representing ∼2.3% of samples in the worst case. The effect of removing such samples on our results was observed to be negligible.

Objective goodness of fit was measured by the G statistic (Sokal and Rohlf 1995, pp. 686–697) and assessed (probabilities estimated) via a parametric bootstrap (Goldman 1993). The null hypothesis says that the alignment is generated by the fitted model with parameter values set at our estimates. The expected site-pattern counts under the model is thus the probability of the pattern multiplied by the alignment length. The alternative is the unrestricted multinomial model, as described in Goldman (1993), taken as the observed site-pattern counts in the alignment. The G statistic is computed from the expected and observed counts using the conventional expression (Sokal and Rohlf 1995, pp. 686–697). The bootstrap procedure is to:

(i) simulate 100 alignments under the null hypothesis of the same length as the fitted alignments;
(ii) perform the original sequential fit on each alignment; and
(iii) calculate the proportion of fitted G statistics that exceed that of the original statistic.

The result is the G statistic parametric bootstrap P-value (hereafter termed the G statistic P-value for succinctness). In every instance we rejected a fit if the G statistic P<0.05.

We evaluated the existence of the molecular clock using likelihood-ratio tests (LRTs) (Gaut et al. 1992). In each case, the null hypothesis was a clock-like model and the alternative was the same model with no constraints on edge length. As such the null model was nested in the alternative, which has one additional degree of freedom. The distribution of the test statistic was assumed to be sufficiently close to a $\chi^{2}$ distribution with one degree of freedom.

The paralinear distance was calculated using our implementation of (4), which is available in the latest version of PyCogent. In all instances, the paralinear distance was calculated directly from the alignments, using empirical joint probability distribution matrices. It was not calculated from any fitted model.

We expected the distances measured using stationary models on data generated by a nonstationary process to exhibit a bias. We further expected the magnitude of this bias to increase with increasing departure from stationarity. We measured the difference in nucleotide composition between two sequences using the Jensen–Shannon divergence (JSD) (Lin 1991). The JSD is an information theoretic measure of distance between probability distributions that is symmetric and extends naturally to multiple distributions. We exclusively employ the equally weighted JSD, defined as

$$\text{JSD}=\text{H}\left(\frac{1}{n}\sum_{i=1}^{n}p_{i}\right)-\frac{1}{n}\sum_{i=1}^{n}\text{H}(p_{i})$$

for n probability mass functions $p_{i}$ defined on the same state space S, where H is the Shannon entropy. We have implemented the Shannon entropy using the natural logarithm as

$$\text{H}(p)=-\sum_{s\in S}p(s)\ln p(s).$$

In our context, S={A,C,G,T}, n is the number of sequences in question (two or three), and p(s) is estimated using the nucleotide frequencies for each sequence.

The JSD allows us to explore an interesting property of the General model. It is not difficult to show that for a nonstationary process, the JSD between f(0) and f(t), that is the distribution of states at an ancestor node and the distribution of states at a point in time t as one moves along a edge, must be a monotonically increasing function of time. This leads us to hypothesize that for similar alignments that exhibit nonstationarity, departure from compositional homogeneity between sequences should be positively correlated with genetic distance. Equally, for alignments that are well explained by stationary processes, we should expect low empirical JSD.

We used R version 3.1.1 (R Core Team 2013) to calculate local regressions (or LOESS fits), regressions through the origin, quantile regressions (Koenker 2013), quantiles, and likelihood-ratio P-values. Plots were prepared using ggplot2 (Wickham 2009) in R. The implementation of the clock-like General model required the use of a numerical root finder for which we used SciPy version 0.14.0 (Jones et al. 2001) in Python.

Calculation of ENS requires the integration of the matrix exponential (2). There are several ways to approach this calculation, but a simple cookbook-style algorithm is to define

$$C=\left(\begin{array}{cc} Q & -\text{diag}(Q)\\ 0^{1\times 4} & 0 \end{array}\right).$$

The ENS is then given by

$$n(t)=f(0){\left(\begin{array}{cccc} \exp{\left(Ct\right)}_{1,5} & \exp{\left(Ct\right)}_{2,5} & \exp{\left(Ct\right)}_{3,5} & \exp{\left(Ct\right)}_{4,5} \end{array}\right)}^{\text{T}},$$

where exp(Ct) is again the matrix exponential that can be calculated by several standard software packages, for instance SciPy. This approach is a simplification of the methods presented in Van Loan (1978). It is not necessarily the fastest algorithm, depending on context, but it is included here for ease of exposition and because it is as robust as matrix exponentiation. Code to calculate n(t) is available in the latest version of PyCogent.

All scripts used for the analyses reported in this manuscript can be downloaded from the Dryad data repository (http://doi:10.5061/dryad.g7g0n).

## Data Sets

All protein coding sequences were sampled from Ensembl release 68. One-to-one orthologs for nuclear and mitochondrial genomes were obtained for human (*Homo sapiens*), mouse (*Mus musculus*), and opossum (*Monodelphis domestica*) using the database querying capabilities of PyCogent (Knight et al. 2007). The sequences were aligned using the PyCogent built-in codon aligner, employing the Muse and Gaut (1994) substitution model. For nuclear encoded sequences, we selected third codon position sites only. Aligned columns with ambiguity or gap characters were excluded from analysis. Alignments were filtered by length to contain at least 500 sites with unambiguous and nongap characters. For mitochondrial encoded genes, all codon positions were used, as these genes are too short for reliable estimation using third codon positions alone. After filtering by length, 4150 nuclear and eight mitochondrial alignments remained.

To establish the relationship between sequence composition and estimation of evolutionary time, we sampled a molecular marker widely employed for studying microbial diversity. An alignment of 408,135 16S rRNA sequences was downloaded from http://www.secondgenome.com/go/2011-greengenes-taxonomy/. The alignment consisted of positions that are conserved in secondary structure (McDonald et al. 2012). As per the filtering of protein coding sequences, aligned positions were excluded if they contained an IUPAC ambiguity or gap character. A sample of 9854 triads was taken. The empirical probability distribution of JSD of randomly chosen triads is concentrated near zero. To test the effect of varying JSD, triads were chosen via a heuristic, pseudorandom algorithm with the goal of producing a sample of triads for which the JSD was roughly uniformly distributed. As a side effect, the triads that were selected were ultimately formed from only 9339 sequences. After filtering by length, 9702 microbial alignments remained.

## Results

### Measurements of Model Fit

We argue that if an evolutionary model adequately explains a data set, then inferences drawn from that model are more likely to be robust than if this condition is not met. Additionally, when a model accounts for the data well then a more complicated model is unnecessary. We assessed whether an alignment was plausibly generated by a model using a goodness-of-fit statistic; comparing the expected distribution of site patterns from the ML model with the observed counts. The probability that an alignment was generated by the fitted model was assessed using the parametric bootstrap with 100 replicates, as described in the Methods section.

**Figure 3.**
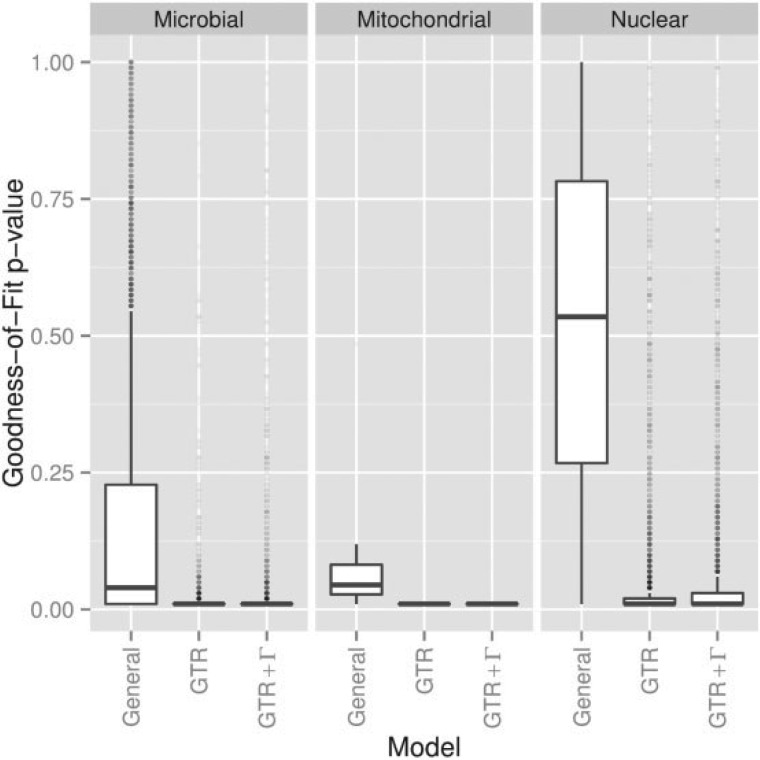
The General model fits empirical data much better than the GTR and General Time-Reversible with rate heterogeneity across sites (GTR+Γ) models, and well in some instances. Box plot shows empirical distributions of G statistic parametric bootstrap P-values by model for 4135 Nuclear, eight Mitochondrial, and 9702 Microbial samples.

In all cases considered, the General model was better able to explain the data than the GTR and GTR+Γ models. The box plots in Figure 3 give a graphical representation of the G statistic P-value distributions for the three data sets and three models. If a null hypothesis is correct, the P-value should be drawn from a uniform distribution, implying that 5% of the tests should produce P<0.05. The proportion of tests producing P-values above that level are shown in Table 1. By this measure the performance of the GTR+Γ model is similar to that of the GTR model, and the General model clearly fits the data better. For the nuclear data, the null hypothesis rejection rate is very similar to the type I error rate. In the analysis that follows, we will focus on cases where the General model plausibly explains the data, so we restrict the data sets to samples where the G statistic P-value for the General model exceeded 0.05. For readers interested in how robust our findings are to admitting alignments that fail this criterion, we have reproduced all of our plots for the alignments where the G statistic P-value for the General model was <0.05 in the online appendix available as Supplementary Material on Dryad at http://dx.doi.org/10.5061/dryad.g7g0n.

**Table 1.** Rate at which model fits failed to be rejected at 5% significance

	General (%)	GTR (%)	GTR+Γ (%)
Nuclear	94	18	20
Mitochondrial	38	0	0
Microbial	47	1.2	2.6

Percentages given to two significant figures.

It is common in a model selection framework to use the Akaike Information Criterion (AIC) (Burnham and Anderson 2002, pp. 60–64) to select between competing models. For reference, we mention that for the data reasonably explained by the General model, under the AIC, GTR+Γ would have been selected ahead of GTR for ∼23% of the 3906 nuclear alignments and ∼77% of the 4557 microbial alignments.

Also common in model selection is the notion of a bias versus variance tradeoff (Burnham and Anderson 2002, pp. 31–35). In our case, we would expect the General model to give less biased estimates of genetic distance than GTR+Γ or GTR, but we would expect to pay for this accuracy with more variable estimates. Similarly, estimates from GTR+Γ should be less biased, but more variable, than those from GTR. We measured the standard deviation (SD) of $d_{\text{ENS}}$, $d_{\text{GTR}}$, and $d_{\text{GTR}+\Gamma }$ from human to mouse using the nuclear data. Over the alignments reasonably explained by the General model, the estimates of the SD were ∼0.10, ∼0.12, and ∼0.18, respectively. These estimates of SD confound the natural variation in the substitution processes between genes with the fitting error that we seek to measure. Given that each substitution model was fitted to the same genes, the contribution of natural variation to the SDs is the same in each case. Accordingly, the different SDs indicate that the General model either exhibited smaller fitting error than the GTR or GTR+Γ models, or that the fitting error for the General model was less correlated with the natural variation than for the GTR or GTR+Γ models. Either contingency favors selection of the General model. Although this result violates the general rule of the bias-variance tradeoff, it is not without precedent. Guindon (2013) reported another example of a parameter-rich evolutionary substitution model that provides more precise parameter estimates than a simpler, nested model.

### Reversible and Stationary Models Overestimate Time

**Figure 4.**
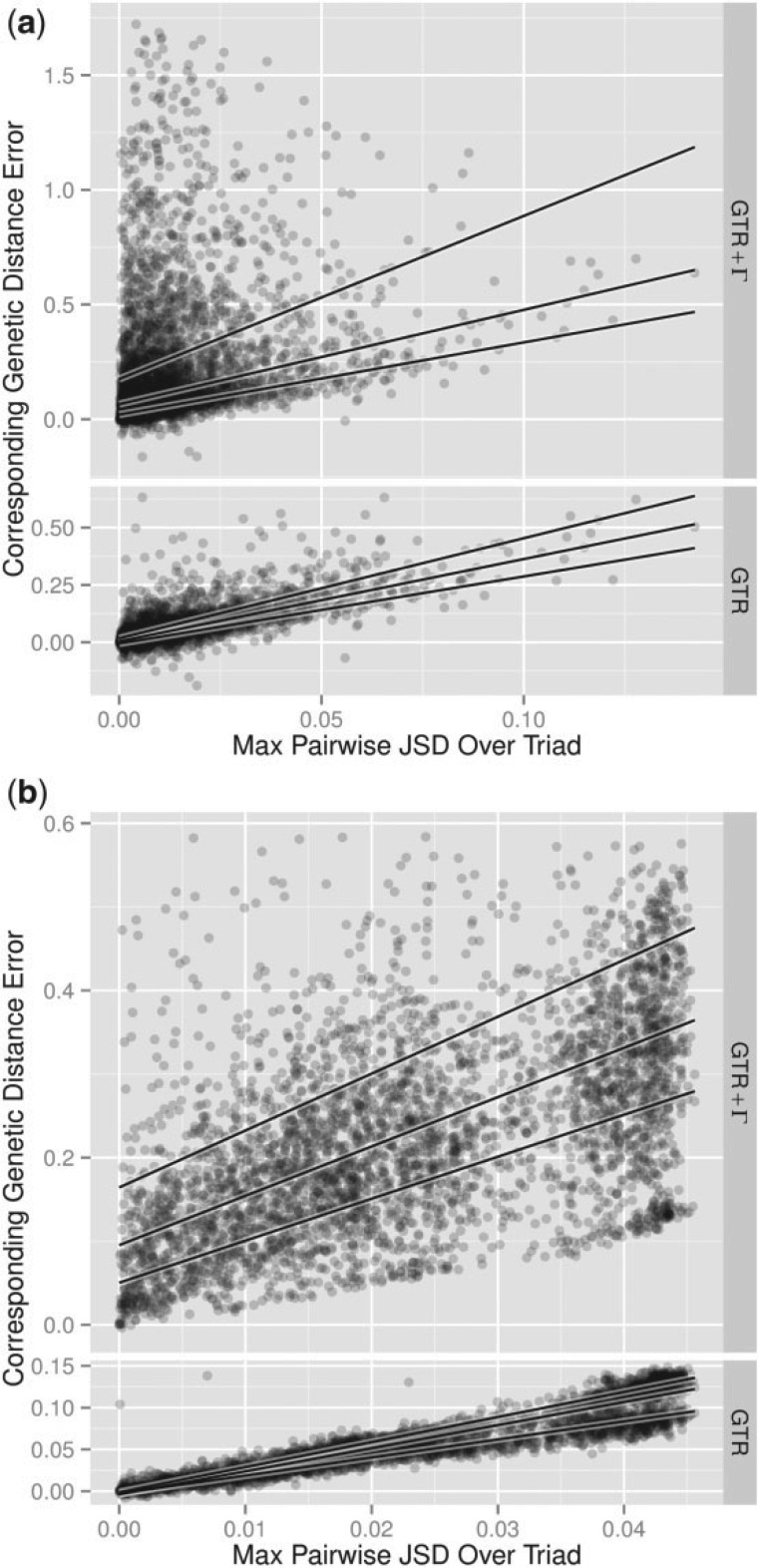
The genetic distance error increases with JSD. Genetic distance is the expected number of substitutions, denoted $d_{\text{ENS}}$, $d_{\text{GTR}}$, and $d_{\text{GTR}+\Gamma }$ as estimated using the General, GTR and GTR+Γ models, respectively. Scatter plots show an empirical relationship between JSD and $d_{\text{GTR}+\Gamma }-d_{\text{ENS}}$ or $d_{\text{GTR}}-d_{\text{ENS}}$. In every case the GTR and GTR+Γ models tend overwhelmingly toward overestimation. Solid lines show quantile regressions for 25%, 50%, and 75% quantiles. All General model fits have goodness-of-fit (G statistic) P-value ≥0.05. a) 3906 alignments of human, mouse, and opossum protein coding genes. b) 4557 alignments of triads of 16S ribosomal RNA.

We expected that discrepancy between $d_{\text{GTR}+\Gamma }$ or $d_{\text{GTR}}$ and $d_{\text{ENS}}$ would increase with increasing departure from compositional homogeneity. We measured this departure using JSD, a distance measure between the nucleotide frequency distributions. For alignments defined as being consistent with the General model (i.e., G statistic P>0.05), we computed the genetic distance error as $d_{\text{GTR}+\Gamma }-d_{\text{ENS}}$ and $d_{\text{GTR}}-d_{\text{ENS}}$. For each alignment we selected the pair of species with maximum JSD, and calculated the genetic distance error between those species. The results are plotted in Figure 4 as a scatter plot with quartile regression lines. In all cases, the genetic distance error is overwhelmingly positive and appears to increase linearly with JSD. The genetic distance error differs between GTR and GTR+Γ primarily in that the latter exhibits larger positive skew, with the conditional interquartile range being at least ∼2.1 times larger for GTR+Γ than GTR in all cases. Additionally, the median regression is steeper for GTR+Γ than for GTR in both cases. We summarize the slopes and intercepts of the median regressions across data sets and models in Table 2. The variation of slopes between data sets is not surprising. Only the third codon position was sampled for the exonic data, in an effort to sample closer to a neutral evolutionary process (Table 2). All of the positions in the microbial data set were used, so some are likely to be affected by natural selection. The difference between the slopes may reflect these underlying differences in the generating processes.

**Table 2.** Median regression results for genetic distance error versus JSD values across data sets and models, as shown in Figure 4

	GTR		GTR+Γ	
	Slope	Intercept	Slope	Intercept
Nuclear	3.6	0.0	4.1	0.1
Microbial	2.8	0.0	5.9	0.1

Results show slopes and intercepts to one decimal place.

**Figure 5.**
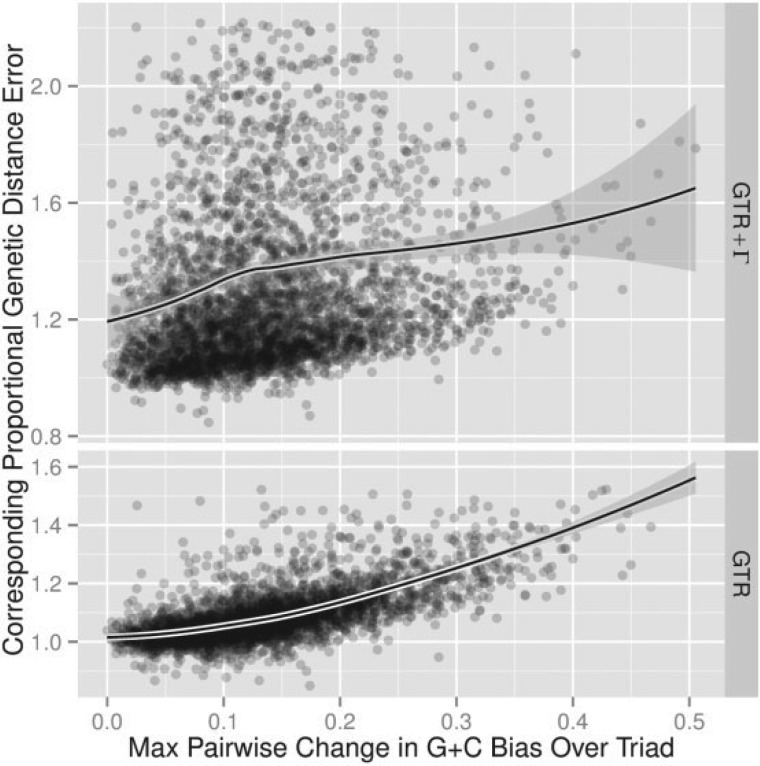
The difference in estimated distances increases with compositional heterogeneity. Scatter plots showing an empirical relationship between the change in G + C content and $d_{\text{GTR}+\Gamma }/d_{\text{ENS}}$ or $d_{\text{GTR}}/d_{\text{ENS}}$. All General model fits have goodness-of-fit P-value ≥0.05. The solid lines show LOESS fits. The plot shows 3906 alignments of human, mouse, and opossum protein coding genes.

It is common to compare the proportion of G + C nucleotides in sequences when discussing composition. For instance, the G + C content at third position in mitochondrial frog DNA is 27.85%, whereas it is 48.57% in chicken (from the codon usage database, Nakamura et al. 2000). Figure 5 shows that $d_{\text{GTR}}/d_{\text{ENS}}$ and $d_{\text{GTR}+\Gamma }/d_{\text{ENS}}$ increase with difference in G + C content in a similar fashion to the trend shown in Figure 4a, but the relationship is not as linear as that between genetic distance error and JSD. For Figure 5 we used the pair of taxa in each triad that displayed maximum difference in G + C content. As the relationship appears to be nonlinear, we have provided LOESS fits in Figure 5. We have also chosen to use JSD as our primary measure of departure from compositional homogeneity because comparing the G + C content between sequences hides changes in compositions that trade Gs for Cs, or vice versa. The following is an indication of the magnitude of the results. For each alignment in our nuclear data set, we took the pair of sequences for which we observed maximal difference in G + C content and calculated $d_{\text{GTR}+\Gamma }/d_{\text{ENS}}$ for this pair. If we then restricted our attention to the 221 alignments for which the difference in G + C content was between 19% and 21% (i.e., roughly the difference between frog and chicken) the average value for $d_{\text{GTR}+\Gamma }/d_{\text{ENS}}$ was ∼1.47. The 2.5% and 97.5% percentiles were ∼1.06 and ∼3.29, respectively.

The differences that we see in the genetic distance error between GTR and GTR+Γ are to be expected: As the GTR model is nested in the General model, GTR and General should yield similar results where the assumptions of GTR are not strongly violated, and depart smoothly in their inferences as the level of contradiction increases. The GTR+Γ and General models are not related in that way, so we see a more consistent bias in the GTR+Γ results across all levels of nonstationarity, and that error increases as one of its key assumptions, stationarity, is violated.

Although the magnitude of the correlation between genetic distance error and nonstationarity varies between data sets and for the two models tested, the tendency for the reversible model to overestimate genetic distance is a consistent property across our data sets and thus putatively the tree of life. Also consistent is that the magnitude of this overestimation reflects the degree of departure from stationarity, that is the extent to which nucleotide composition differs between taxa.

### Departure from Compositional Homogeneity Increases with Genetic Distance

**Figure 6.**
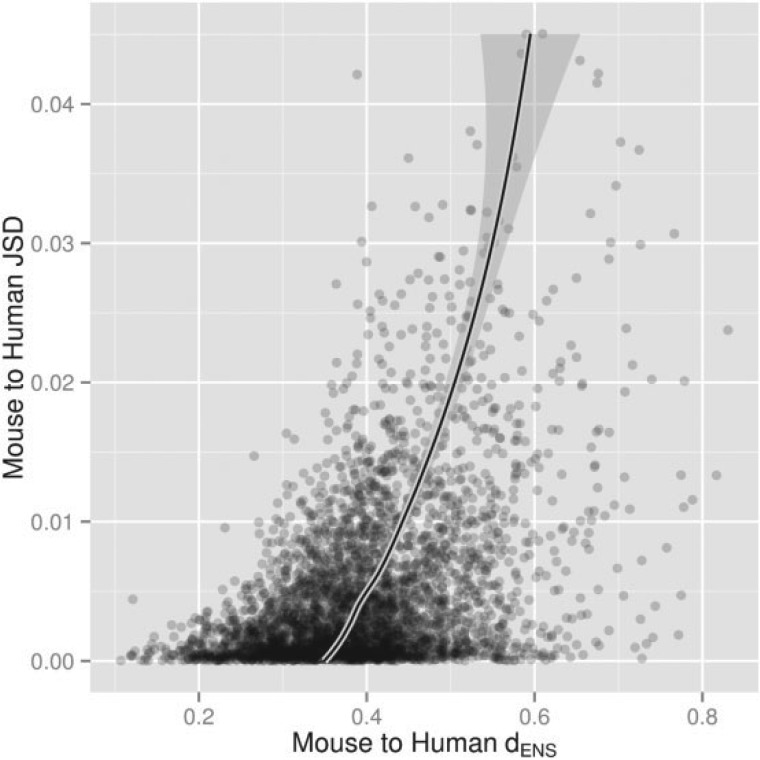
Nonstationarity increases with distance. Scatter plots showing an empirical relationship between JSD and the $d_{\text{ENS}}$ for the human/mouse pair. All General model fits have goodness-of-fit P≥0.05. The solid line shows LOESS fit. The plots show 3906 alignments of human, mouse, and opossum protein coding genes.

In the Methods section, we hypothesized that the nonstationarity between sequences should increase with genetic distance. Figure 6 plots JSD against genetic distance under the General model for the mouse to human path in the nuclear data for alignments plausibly modeled by the General model. We do not postulate the nature of the relationship between $d_{\text{ENS}}$ and JSD, other than that it is increasing, so we provide a LOESS plot of $d_{\text{ENS}}$, as it is expected and observed to be significantly more noisy than JSD. The relationship is observed to be increasing, supporting the hypothesis that sequences that are further apart in a phylogenetic sense tend to display greater compositional heterogeneity. Note that there is a gray area here—tests of compositional heterogeneity will lack power for closely related sequences, so the question really pertains to long edges only.

### Comparison Between ENS and Paralinear Distance

**Figure 7.**
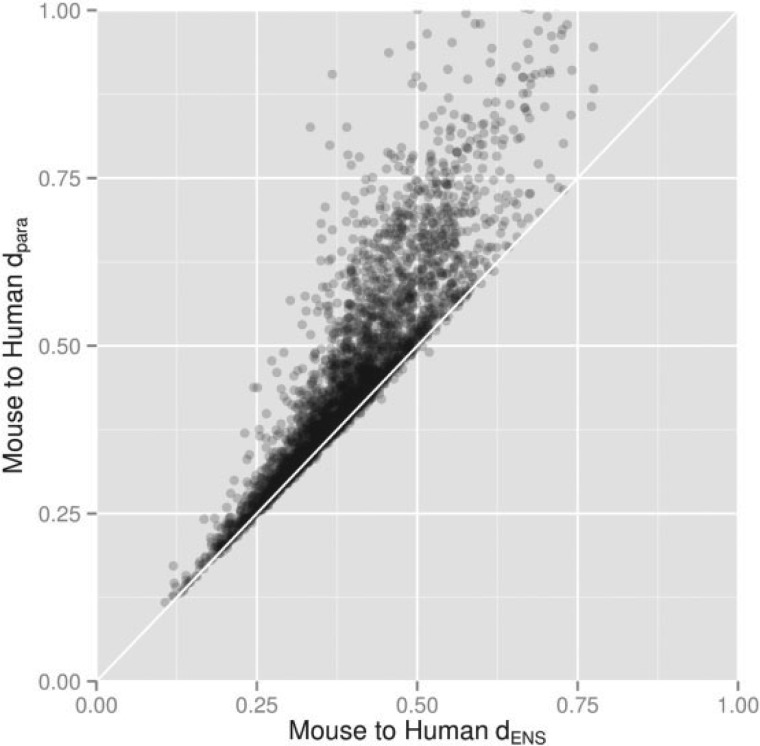
The paralinear distance ($d_{\text{para}}$) exceeds $d_{\text{ENS}}$ in almost all cases. Scatter plot of $d_{\text{para}}$ versus $d_{\text{ENS}}$ between human and mouse from 3906 alignments of opossum, mouse, and human protein coding genes. All General model fits have goodness-of-fit P≥0.05. Straight line shows the diagonal.

Paralinear distances were originally proposed to address the known occurrence of nonstationarity (Lake 1994). Our analysis shows that this distance, $d_{\text{para}}$, is also prone to systematic bias (Fig. 7), and is almost always greater than or equal to $d_{\text{ENS}}$. A linear regression of this data in which the intercept is constrained to be zero has a slope of ∼1.158 with an $R^{2}$ value of ∼0.97, meaning that there is still a strong relationship between $d_{\text{para}}$ and ENS. The 95% confidence interval for the slope was (1.152,1.164).

Purely for comparison, we also give the results for the relationship between $d_{\text{GTR}}$ and $d_{\text{ENS}}$. In this case, a linear regression through the origin yielded a slope with a 95% confidence interval of (1.034,1.040) and an $R^{2}$ of ∼0.99. It is interesting that the GTR model outperforms the paralinear distance in this instance. It would appear that the errors that are introduced by the paralinear approximation are greater than the errors introduced by neglecting the existence of nonstationarity in the data.

### Influence of Substitution Model Choice on the Molecular Clock

We sought to determine whether a fundamental feature, the existence of a molecular clock, was altered by model selection between the GTR or GTR+Γ and General models. We found that departure from a molecular clock was overstated under the GTR model, and more so under the GTR+Γ model.

**Figure 8.**
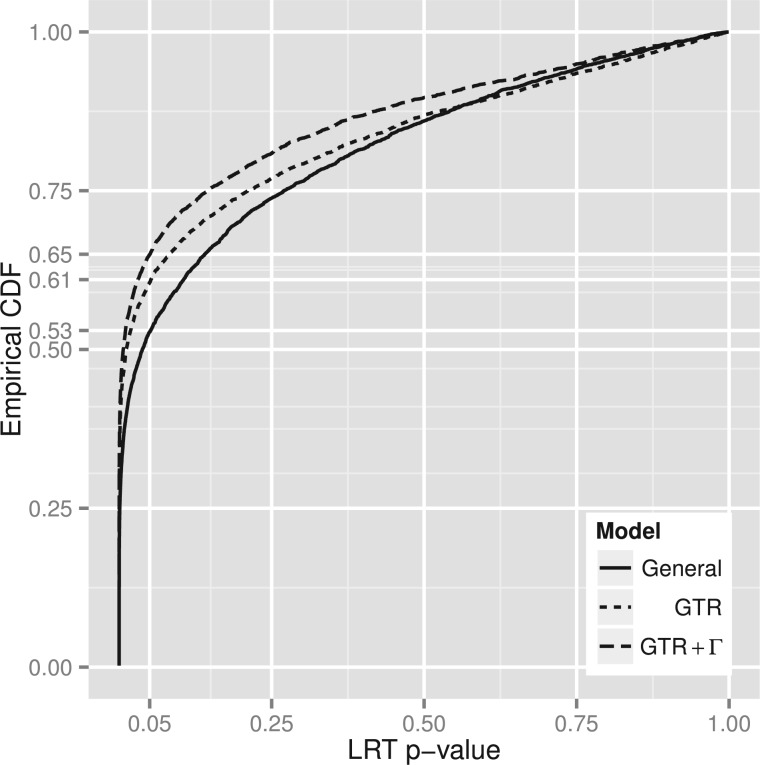
The molecular clock is rejected more often under the GTR and GTR+Γ models. Empirical cumulative distribution functions of likelihood ratio test P-values between constrained clock-like and unconstrained models based on GTR, GTR+Γ, and General models over 3909 alignments of human, mouse, and opossum protein coding genes. All General model fits have goodness-of-fit P≥0.05.

We compared the fit of clock-like and unconstrained models (Methods section) to the nuclear data where the ingroup was specified to contain mouse and human. That is, we tested the existence of a molecular clock on the mouse and human edges. We found that the clock-like model was rejected in favor of the unconstrained model more often by the GTR or GTR+Γ models than by the General model (Fig. 8). For instance, at 5% significance the GTR model rejected the molecular clock in ∼61% of cases and the GTR+Γ model rejected the molecular clock in ∼65% of cases, whereas the General model clock rejection rate was ∼53%. Of 3909 alignments, application of the General model led to the clock being rejected 2062 times (at the nominal 5% level). Of these instances, the mouse branch was longer than the human branch in 2024 cases, consistent with the hypothesis of an accelerated substitution rate on the mouse branch.

**Figure 9.**
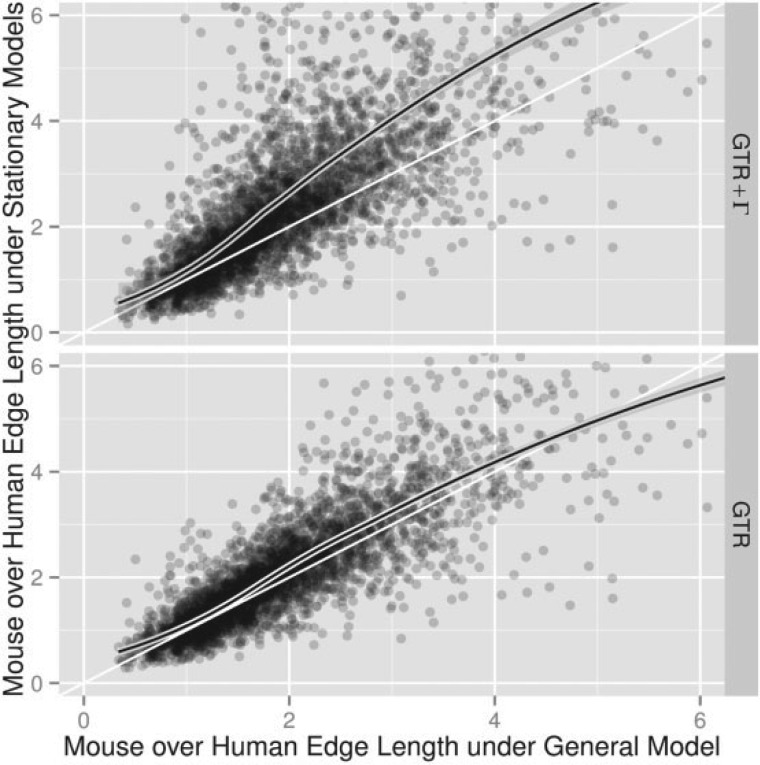
GTR and GTR+Γ models overestimate departures from the molecular clock. Scatter plots of the ratio of mouse and human edge lengths measured as $d_{\text{GTR}}$ or $d_{\text{GTR}+\Gamma }$ against $d_{\text{ENS}}$ from 3906 alignments of human, mouse, and opossum protein coding genes. All General model fits have goodness-of-fit P≥0.05. Plots were truncated near six; distance estimates greater than 100 were rejected as outliers. The curved lines show LOESS fits. Straight lines show the diagonal.

Figure 9 illustrates a more direct comparison, where we plot the ratio of $d_{\text{ENS}}$ between mouse and human edges to the ratio of $d_{\text{GTR}}$ and $d_{\text{GTR}+\Gamma }$ between mouse and human edges. There is a tendency for the length of the mouse edge to exceed that of the human edge by a greater amount under both the GTR and GTR+Γ models. In other words, the speedup of substitution rates on the mouse lineage is overstated by the stationary models.

The results from our analysis of the properties of a molecular clock show that the systematic overestimation of genetic distance by GTR and GTR+Γ can effect different edges to different extents. This overestimation was shown to be stronger on the mouse edge than the human edge, resulting in a different level of inference being drawn about a fundamental quality of the data. The effect was found to be stronger for GTR+Γ than for GTR. Specifically, the estimated ratios of mouse/human substitution rates and 95% confidence intervals were: General 1.92 (1.89, 1.95); GTR 2.08 (2.04, 2.12); GTR+Γ 2.49 (2.42, 2.57). Employing a time-reversible, rate-heterogeneous model would, therefore, be expected to cause an overestimation of the ratio of rodent to primate evolutionary rate by ∼1.3-fold.

## Discussion

Acknowledging that all models are wrong, we have sought to identify ways in which the prevailing nucleotide substitution models in use by the phylogenetics community might be importantly wrong (in the sense used by Box 1976). We have given particular attention to the GTR model, as the most general model in popular use, and its extension, GTR+Γ. Across our spectrum of tests, we found that neither model explains evolutionary processes very well, and that in comparison to a model that can (the General model), application of the GTR or GTR+Γ model leads to incorrect fundamental inferences. We also assert that use of the General model, which is the most general identifiable time-homogeneous continuous-time nucleotide substitution model, in their stead is practical and sensible.

We separately and objectively assessed the goodness of fit of the GTR, GTR+Γ, and General models across samples of nuclear, mitochondrial, and microbial genes. Where the GTR and GTR+Γ models performed best, for the nuclear sample, they were rejected as the possible generating process more than 80% of the time at 5% significance. We are not the first authors to observe that a GTR model fails parametric bootstrap tests (Jayaswal et al. 2007). For the same data set, the G statistic P-values for the General model were roughly uniformly distributed, indicating that it could plausibly be the generating process for this data. For the mitochondrial and microbial genes, the General model failed to be rejected at least an order of magnitude more often than the GTR and GTR+Γ models. We do not contend that any of our data sets were necessarily generated by a process that satisfies the assumptions of the General model, only that it is plausibly the generating process in some instances. Further investigation of models of nucleotide substitution that relax the General model assumption that sites evolve identically and independently may be justified.

Under the GTR or GTR+Γ models and in comparison with the General model, one is led to overstate genetic distances for nuclear and microbial sequences, overstate how frequently the molecular clock assumption is violated by mouse and human evolution, and overstate the ratio of mouse to human phylogenetic edge lengths. Importantly, the extent to which distances are overstated was found to be proportional to the change in nucleotide composition between sequences. We have also found a theoretical basis and empirical evidence for the notion that longer edges are more likely to exhibit greater compositional heterogeneity. It is, therefore, easy to postulate that under the GTR or GTR+Γ models the worst effect of overestimation will be evident in long edges. This effect is reminiscent of the classic problem of long branch attraction that is associated with parsimony methods. Note that long branch attraction is also known to manifest in ML approaches where the model is not supported by the data (Bergsten 2005).

Our results have important implications for published and future studies concerning molecular clocks. Although we confirmed substitution rate acceleration on the rodent lineage, our results indicate that the choice of a time-reversible model can markedly increase estimates of the magnitude of acceleration. Our results further established a greater tendency to reject the clock under time-reversible models. More troubling is the observation that under the commonly employed GTR+Γ model, the average rodent substitution rate increase was ∼1.3-fold larger. In contrast, estimates from GTR alone were much closer to those from the General model. The tendency of time-reversible models toward overestimation of edge lengths, particularly for long edges, is a plausible contributor to the tendency for molecular clock based estimates of divergence time to be older than those from the fossil record (Blair Hedges and Kumar 2003). This potential was noted earlier by Kumar and Subramanian (2002) through elimination of sequences based on tests for nonstationarity (Kumar and Gadagkar 2001; Kumar and Subramanian 2002). Our results predict that using $d_{ENS}$ for estimation of divergence times will improve reliability of those estimates and conceivably reduce their discrepancy with geologically based dates.

It is tempting to infer that the General model will not give markedly different distance estimates to the GTR model when a measure of nonstationarity, such as JSD or the disparity index (Kumar and Gadagkar 2001), is small. However, it is possible that sequences that are not significantly compositionally different may still have been generated by processes that were not reversible, which could again lead to bias that would not be present under the General model. Evidence for nonreversible, stationary evolutionary processes has been reported (Jayaswal et al. 2005). We have not pursued this line of enquiry here.

We also tested a pairwise distance measure that is intended for use in instances where the stationarity assumption is violated, the paralinear distance, and found that it, too, systematically overestimates distances in comparison to those implied by the General model fits to the nuclear data.

It is surprising that the bias introduced by using the GTR or GTR+Γ models or paralinear distance to measure genetic distance rather than the General model is consistently positive. This is an empirical result, not a manifestation of a mathematical principle. Figure 4 presents plentiful examples of distances below the diagonal, indicating $d_{ENS}$ is greater than $d_{\text{GTR}}$ or $d_{\text{GTR}+\Gamma }$. We also showed in the Methods section that naïve application of (1), which gives the expected number of substitutions in the stationary case, can over or underestimate the true number of expected substitutions in the nonstationary case. Guindon (2013) showed via simulation that a bias exists between two extensions of the model of Hasegawa et al. (1985). The two extensions both allowed the rate matrix Q to vary along edges, one deterministically and the other stochastically. However, direct comparison between those results and those presented here is made difficult by significant differences in modeling assumptions and experimental setup.

It is widely held that the GTR model underestimates genetic distance in comparison to its rate-heterogeneous extensions (see Waddell and Steel 1997 and references therein). However, we note that in comparison to the General model, the GTR model performed better than the GTR+Γ model in all of our genetic distance and molecular clock tests. That is, biases were greater and the molecular clock hypothesis rejected more frequently under GTR+Γ. This result is counterintuitive because use of the AIC would lead one to select GTR+Γ ahead of GTR for modeling the majority of our microbial and a substantial proportion of our nuclear data. If the General model is excluded from the comparison, taking just AIC or maximized log-likelihood values as a guide, one might be tempted to confirm the results in Waddell and Steel (1997). We argue that the community has been misled by these sorts of results. Although accommodating rate heterogeneity may be justified, our results suggest that it is far more important to relax the assumption of stationarity.

Although our goodness-of-fit results establish the merits of the General model, we have not considered its suitability for the phylogenetic reconstruction problem. We previously demonstrated from a survey across a diverse array of lineages that, for the vast majority of considered cases, the BH and General models were equivalent (Verbyla et al. 2013). Accordingly, the statistical advantages and pitfalls of the General model for the phylogenetic reconstruction problem correspond to those already identified for BH (e.g., Jayaswal et al. 2005, 2007). In both cases, a major barrier to application of these models for full tree-space searches is the large number of free parameters. Application to phylogenetic reconstruction of even modest numbers of taxa will require attention be paid to fast approximation techniques.

With advances in software and hardware and the prodigious amount of genetic information available online, it is now possible and practical to measure distances using the parameter rich General model, as we have demonstrated. An important property of distances measured under the General model is that they reduce to those measured under the GTR model, and all its submodels, should the data at hand reflect the assumptions of GTR or its submodel. From this perspective we see no reason to use the GTR model to estimate distances. Any remaining concerns about performance could be addressed by approximation techniques or potentially by adding constraints to the General model that do not impede its ability to fit the data, although we have not explored that here.

Our analyses strongly indicate that when estimating genetic distances, stationary, time-reversible models should be avoided. The widely employed model selection approaches select from a collection of similarly violated processes. We have shown that as evolutionary processes they fail to plausibly explain available observations and add a positive bias of a magnitude that varies by phylogenetic edge to genetic distance estimates. In our view, it is difficult to justify their continued use when a better alternative exists, which yields the same estimates as any submodel, including GTR, if its assumptions are justified.

## Supplementary Material

Data available from the Dryad Digital Repository: http://dx.doi.org/10.5061/dryad.g7g0n.
